# Supplementary material for: Methylene Blue-Loaded Mesoporous Silica-Coated Gold Nanorods on Graphene Oxide for Synergistic Photothermal and Photodynamic Therapy
Source: Pharmaceutics. 2022 Oct 20;14(10):2242. doi: 10.3390/pharmaceutics14102242 (PMC9612258; doi:10.3390/pharmaceutics14102242)
Supplement: Supplementary file 1 [file pharmaceutics-14-02242-s001.zip › pharmaceutics-1907615-supplementary.pdf]

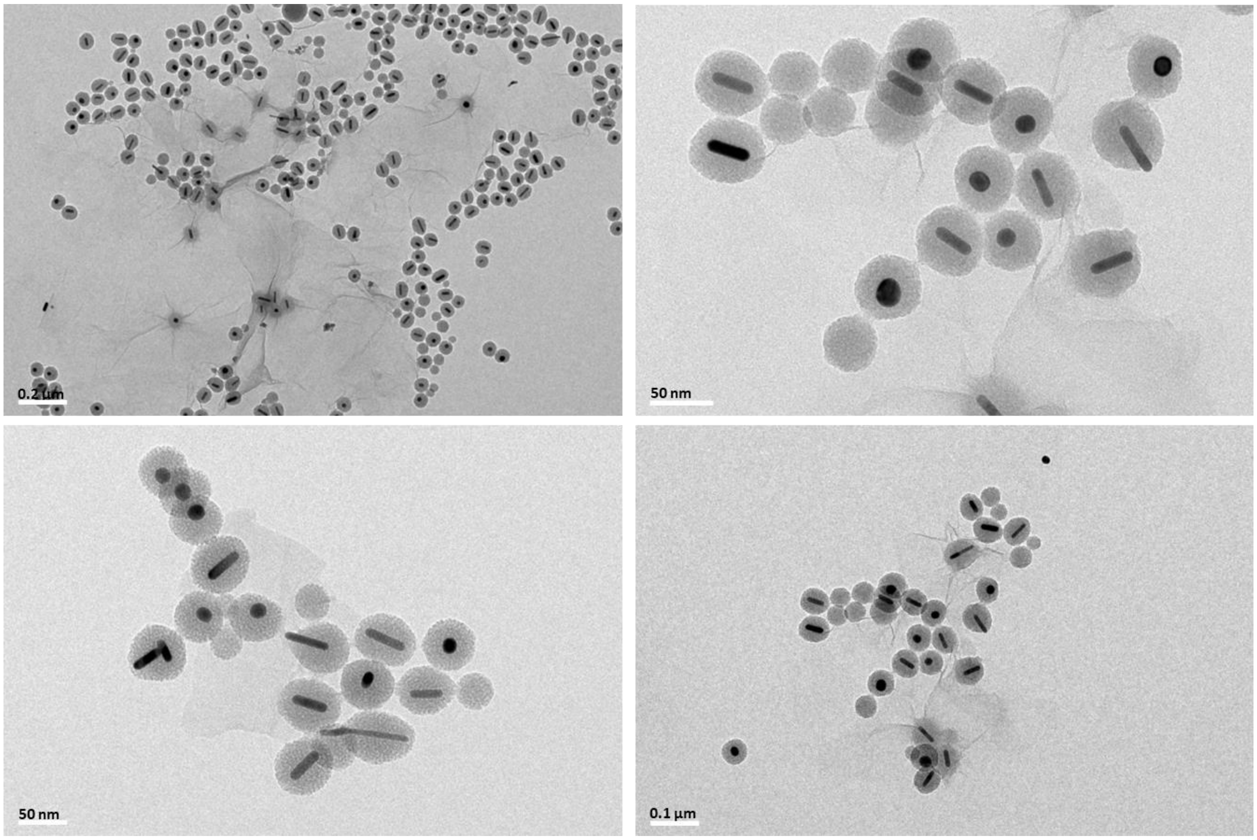

Figure S1. FETEM images of methylene blue-loaded mesoporous silica-coated gold nanorods on graphene oxide (MB-GNR@mSiO_2-GO).

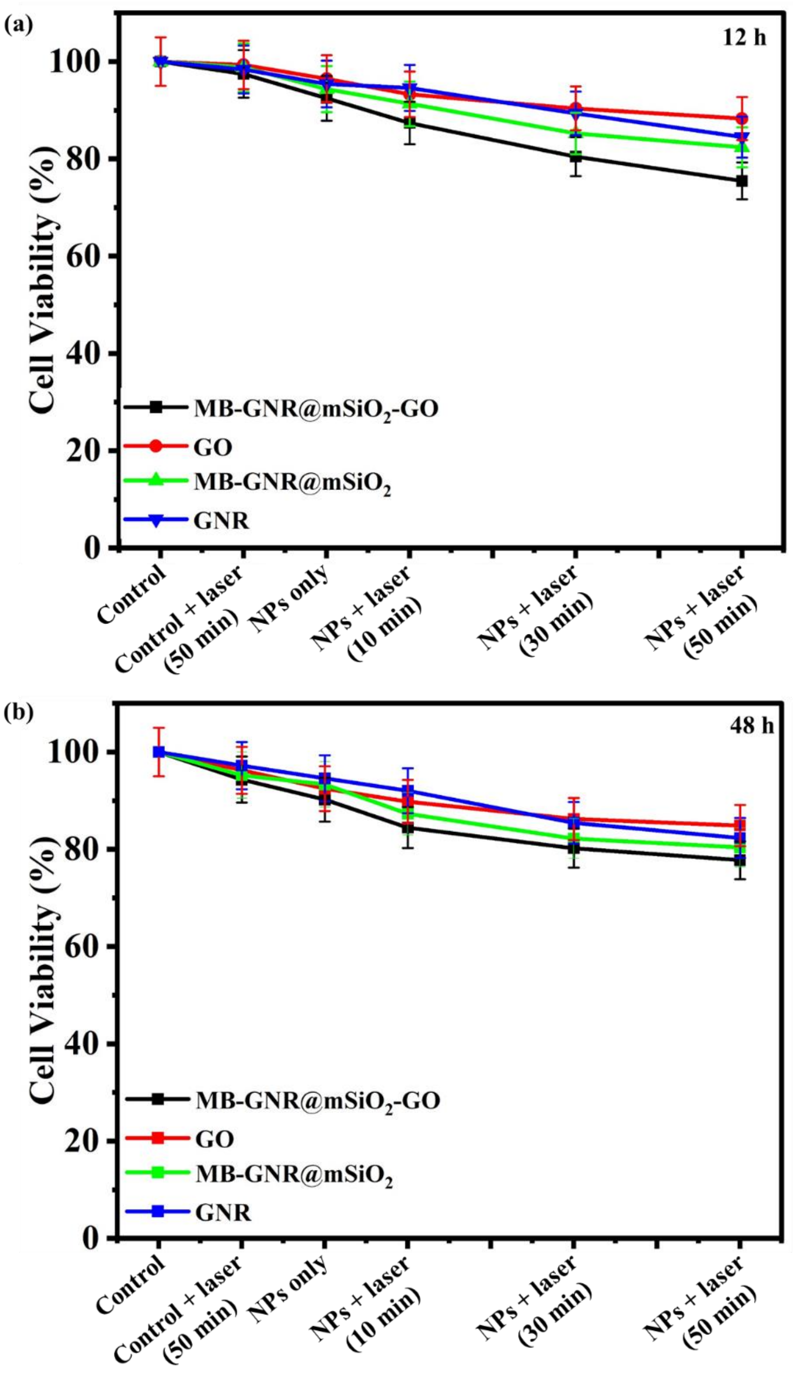

Figure S2. Viability of BLO-11 cells treated with gold nanorod (GNR), methylene blue-loaded mesoporous silica-coated gold nanorod (MB-GNR@mSiO_2), graphene oxide (GO), and methylene blue-loaded mesoporous silica-coated gold nanorods on graphene oxide (MB-GNR@mSiO_2-GO) (200 μL/mL) for 12 h (a) and 48 h (b) with or without 785 nm laser irradiation at 0.8 W/cm^2 for various time points (0, 10, 30, and 50 min). Data were expressed as mean ± S.D (n = 3).

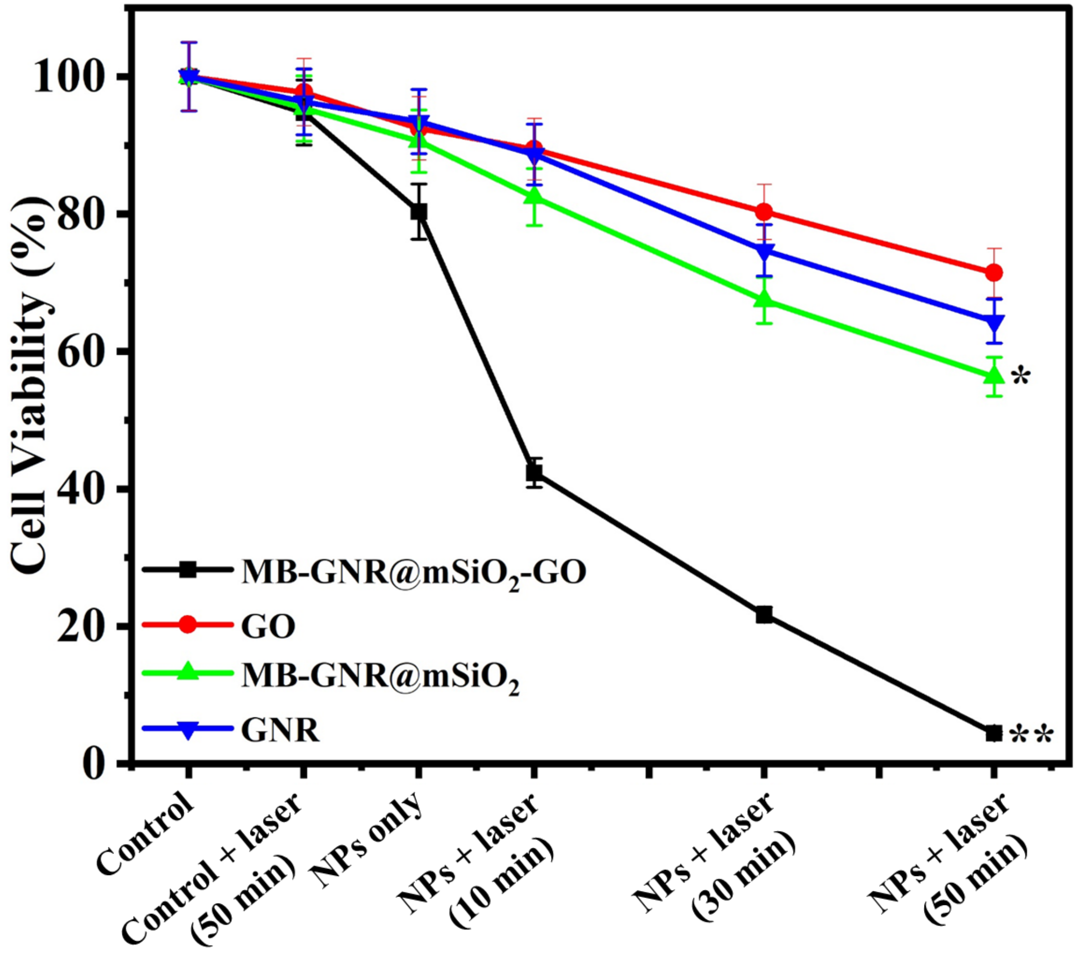

Figure S3. Viability of MDA-MB-231 cells treated with gold nanorod (GNR), methylene blue-loaded mesoporous silica-coated gold nanorod (MB-GNR@mSiO_2), graphene oxide (GO), and methylene blue-loaded mesoporous silica-coated gold nanorods on graphene oxide (MB-GNR@mSiO_2-GO) (200 μL/mL) for 48 h with or without 785 nm laser irradiation at 0.8 W/cm^2 for various time points (0, 10, 30, and 50 min). Data were expressed as mean ± S.D. (n = 3, * significant $P < 0.05$, ** significant $P < 0.01$).
